# Supplementary material for: Mental health disorders, and associated factors among children aged 6–17 years living in Mahama refugee camp in Rwanda
Source: PLOS Ment Health. 2026 Apr 10;3(4):e0000568. doi: 10.1371/journal.pmen.0000568 (PMC13068332; doi:10.1371/journal.pmen.0000568)
Supplement: S1 Table — (DOCX) [file pmen.0000568.s001.docx]

**S1 Table**: Distribution of sociodemographic factors and Food security across Depression, PTSD and suicidal ideation

| **Variable** | **All participants (%)** | **Depression** | | **PTSD** | | **Suicidal ideation** | |
| --- | --- | --- | --- | --- | --- | --- | --- |
|  |  | Yes  63 (12.6%) | p-value | Yes  15 (3.0%) | p-value | Yes  11 (2.2%) | p-value |
| Age; median (IQR) | 12 (10-14) | - | **<0.001** | - | **0.042** | - | **0.032** |
| Gender |  |  |  |  |  |  |  |
| Male | 222 (44.4) | 19 (30.2) | **0.010** | 6 (40.0) | 0.734 | 1 (9.1) | **0.017** |
| Female | 278 (55.6) | 44 (69.8) |  | 9 (60.0) |  | 10 (90.9) |  |
| Nationality |  |  |  |  |  |  |  |
| Burundian | 425 (85.0) | 43 (69.4) | **<0.001** | 8 (53.3) | **<0.001** | 5 (45.4) | **<0.001** |
| Congolese | 75 (15.0) | 20 (26.7) |  | 7 (46.7) |  | 6 (54.6) |  |
| Orphan status |  |  |  |  |  |  |  |
| Have both parents | 394 (78.8) | 40 (64.5) | **0.004** | 9 (60.0) | 0.072 | 6 (54.6) | 0.059 |
| Lacking at least one parent | 106 (21.2) | 22 (35.5) |  | 6 (40.0) |  | 5 (45.4) |  |
| Disability |  |  |  |  |  |  |  |
| Yes | 17 (3.4) | 5 (7.9) | **0.031** | 2 (13.3) | **0.031** | 3 (27.3) | **<0.001** |
| No | 483 (96.6) | 58 (92.1) |  | 13 (86.7) |  | 8 (72.7) |  |
| Food insecurity: *Median* (IQR) | 12 (2-18) | - | **<0.001** | - | **0.020** | - | **0.038** |
