# Supplementary figures and images for: Mental health disorders, and associated factors among children aged 6–17 years living in Mahama refugee camp in Rwanda
Source: PLOS Ment Health. 2026 Apr 10;3(4):e0000568. doi: 10.1371/journal.pmen.0000568 (PMC13068332; doi:10.1371/journal.pmen.0000568)

S1 Fig: Bar graph representing number of households per category of food insecurity.


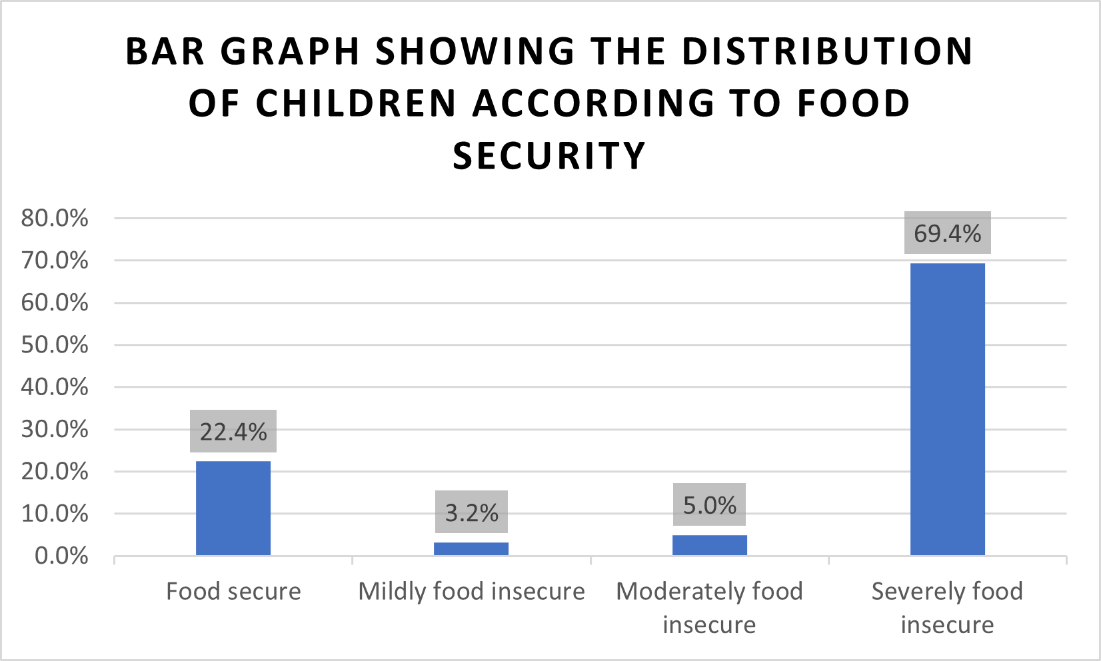

Supplement: S1 Fig — (DOCX) [file pmen.0000568.s002.docx]
