# Supplementary material for: Mental health disorders, and associated factors among children aged 6–17 years living in Mahama refugee camp in Rwanda
Source: PLOS Ment Health. 2026 Apr 10;3(4):e0000568. doi: 10.1371/journal.pmen.0000568 (PMC13068332; doi:10.1371/journal.pmen.0000568)
Supplement: S2 Table — (DOCX) [file pmen.0000568.s003.docx]

**S2 Table**: Multivariate logistic regression table of Depression, PTSD, suicidal ideation and associated factors

| **Variable** | **Depression** | | **PTSD** | | **Suicidal ideation** | |
| --- | --- | --- | --- | --- | --- | --- |
|  | aOR (95% CI) | p-value | aOR (95% CI) | p-value | aOR (95% CI) | p-value |
| Food insecurity | 1.12 (1.07-1.17) | **<0.001** | 1.07 (0.99-1.15) | 0.096 | 1.05 (0.96-1.15) | 0.275 |
| Age | 1.26(1.13-1.42) | **<0.001** | 1.22(0.13-1.50) | 0.060 | 1.20(0.93-1.54) | 0.153 |
| Gender |  |  |  |  |  |  |
| Male | 0.66(0.35-1.26) | 0.208 | 1.31(0.42-4.13) | 0.646 | 0.19(0.02-1.57) | 0.123 |
| Female | Ref |  | Ref |  | Ref |  |
| Nationality |  |  |  |  |  |  |
| Burundian | Ref |  | Ref |  | Ref |  |
| Congolese | 2.83(1.38-5.79) | **0.005** | 4.83 (1.55-15.04) | **0.007** | 6.91(1.79-26.69) | **0.003** |
| Orphan status |  |  |  |  |  |  |
| I have both parents | Ref |  | Ref |  | Ref |  |
| Lacking at least one parent | 1.42 (0.72-2.78) | 0.307 | 1.19 (0.36-3.92) | 0.773 | 1.38 (0.34-5.64) | 0.653 |
| Disability |  |  |  |  |  |  |
| Yes | 2.15 (0.62-7.43) | 0.227 | 4.29 (0.78-23.73) | 0.095 | 9.44 (1.83-48.77) | **0.007** |
| No | Ref |  | Ref |  | Ref |  |
