# Supplementary material for: Mental health disorders, and associated factors among children aged 6–17 years living in Mahama refugee camp in Rwanda
Source: PLOS Ment Health. 2026 Apr 10;3(4):e0000568. doi: 10.1371/journal.pmen.0000568 (PMC13068332; doi:10.1371/journal.pmen.0000568)
Supplement: S3 Table — (DOCX) [file pmen.0000568.s004.docx]

**S3 Table:** Bivariate logistic regression of depression, PTSD, and suicidal ideation and associated factors among children aged 6–17 years in Mahama refugee camp, Rwanda.

| **Variable** | **Depression** | | **PTSD** | | **Suicidal ideation** | |
| --- | --- | --- | --- | --- | --- | --- |
|  | OR (95%CI) | p-value | OR | p-value | OR | p-value |
| Food insecurity | 1.13 (1.08-1.17) | **<0.001** | 1.09 (1.01-1.17) | **0.024** | 1.09 (1.002-1.19) | **0.044** |
| Age | 1.30 (1.17-1.44) | **<0.001** | 1.23(1.01-1.49) | **0.035** | 1.27(1.01-1.60) | **0.042** |
| Gender |  |  |  |  |  |  |
| Male | 0.47 (0.26-0.84) | **0.011** | 0.83(0.29-2.38) | 0.735 | 0.12(0.02-0.96) | **0.046** |
| Female | Ref |  | Ref |  | Ref |  |
| Nationality |  |  |  |  |  |  |
| Burundian | Ref |  | Ref |  | Ref |  |
| Congolese | 3.07 (1.67-5.64) | **<0.001** | 5.45(1.91-15.51) | **0.002** | 7.41(2.20-24.96) | **0.001** |
| Orphan status |  |  |  |  |  |  |
| Not orphan | Ref |  | Ref |  | Ref |  |
| Lacking at least one parent | 2.35 (1.32-4.16) | **0.004** | 2.59 (0.90-7.46**)** | 0.077 | 3.23 (0.97-10.81) | 0.057 |
| Disability |  |  |  |  |  |  |
| Yes | 3.10 (1.06-9.14) | **0.04** | 4.81(1.00-23.24) | 0.051 | 12.70(3.04-53.02) | **<0.001** |
| No | Ref |  | Ref |  | Ref |  |
