# Supplementary material for: Mental health disorders, and associated factors among children aged 6–17 years living in Mahama refugee camp in Rwanda
Source: PLOS Ment Health. 2026 Apr 10;3(4):e0000568. doi: 10.1371/journal.pmen.0000568 (PMC13068332; doi:10.1371/journal.pmen.0000568)
Supplement: Attachment — Submitted filename: PLOS mental health journal addressed comments.docx [file pmen.0000568.s007.docx]

**PLOS mental health journal addressed comments**

1. Please amend your detailed Financial Disclosure statement. This is published with the article. It must therefore be completed in full sentences and contain the exact wording you wish to be published.

Please clarify all sources of financial support for your study. List the grants, grant numbers, and organizations that funded your study, including funding received from your institution. Please note that suppliers of material support, including research materials, should be recognized in the Acknowledgements section rather than in the Financial Disclosure. 
ii. State the initials, alongside each funding source, of each author to receive each grant. For example: "This work was supported by the National Institutes of Health (####### to AM; ###### to CJ) and the National Science Foundation (###### to AM)."
iii. State what role the funders took in the study. If the funders had no role in your study, please state: “The funders had no role in study design, data collection and analysis, decision to publish, or preparation of the manuscript.”
iv. If any authors received a salary from any of your funders, please state which authors and which funders.

**Response:** This has been addressed on page 13 under the funding section

2. Please ensure that your Ethics Statement is available in its entirety at the beginning of your Methods section, under a subheading 'Ethics Statement'.

**Response**: addressed on page 3 in a section headed Ethics Statement under the methods section

3. We notice that your supplementary tables are included in the manuscript file. Please remove them and upload them with the file type 'Supporting Information'. Please ensure that each Supporting Information file has a legend listed in the manuscript after the references list.

**Response:** The supplementary tables have been put in a different document named “Supplementary information”

4. We have noticed that you have uploaded Supporting Information files, but you have not included a list of legends. Please add a full list of legends for your Supporting Information files after the references list.

**Response:** A legends for supplementary tables 1 and 2 have been spared in the results section on pages 7, 8 and 9 whereas a legend for supplementary table 3 has added for the supplementary table in the main manuscript after the references on page 17

5. We note that your Data Availability Statement is currently as follows: “All data generated and analysed during this study are presented in the manuscript”

**Response:** This is among the documents that have been shared

6. Please can you include page and line numbers in a resubmission - this helps facilitate peer review.

**Response**: Page numbers have been added

7. Abstract - please use the full wording for DRC in the abstract on first mention, I presume this is referring to the Democratic Republic of the Congo?

**Response**: Addressed on page 2, 1^st^ paragraph

8. Page 4: "A review done in 2019" could be better phrased - was this a systematic literature review or some other type of review (e.g., review of policy documents, medical records)? Please clarify. A similar point applies on the next page in reference to a 'review done' - a 'review was conducted' would be better wording.

**Response**: Addressed on page 2, last paragraph and page 3, 1^st^ paragraph

9. The introduction frames the project as examining mental health disorders broadly, but the analyses center on three specific outcomes: depression, PTSD, and suicidal ideation. Early sections cite prevalence for depression, PTSD, and suicidal ideation, yet the stated aims revert to generalized references to mental health disorders, creating ambiguity about the study’s scope. To improve clarity and consistency, the authors may consider revising the introduction to clearly state from the outset that these three outcomes are the primary focus of the study and ensure the aims reflect this more specific scope.

**Response:** This has been addressed in the last paragraph of page 3

10. Methods: was there any translation or adaptation of the study materials for the sample? Where there any challenges with literacy with the target sample given their status as refugees?

**Response**: Yes, translation was done to Kinyarwanda, and the data enumerators, with good proficiency in Kinyarwanda and Swahili, were trained to explain the questions to the participants in a similar way to ensure consistency in the way questions were being asked.

11. Results: "Most of them were aged between 10-14 years" - this could be clearer and more specific, i.e. how many were aged between 10-14 years? Could you provide a percentage or raw number here?

**Response**: This has been addressed on page 7 under the Results section, sub-section study participants

12. Food insecurity - it would be useful to have a bit more commentary in the manuscript about how/why the participants had high levels of food insecurity - was this primarily due to their experiences before arriving at the refugee camp and/or were there specific issues with food availability/security in the camp itself?

**Response**: This has been addressed on Page 11 in paragraph 5

13. Table 1 - it could be much clearer what the specific analyses are that are reported in this table - it was not easy to understand what the p values were associated with in the Table or which outcome relates to which analysis.

**Response**: The analysis used was bivariate using chi-square and mann-whitney U tests and those were the associations and their respective p-values in relation to the outcome variables.

14. There also seemed to be remarkably low incidences of PTSD and suicidal ideation in the sample, is this correct?

**Response**: Yes, those were the incidences found among those children

15. Figure 1 - this is also difficult to interpret due to the presentation of both the percentages and raw numbers for each group - is this bar graph necessary or could this information be better presented in the main text?

**Response**: That has been addressed on page 7. The text description has been spared and the graph has been erased as requested

16. As a non-African researcher based in Europe, I found it difficult to unpack why the 'DRC' was a key factor in the regression models - I am presuming that it is less about refugees coming from this specific country and more about instability and possible trauma/fighting in the DRC that is key? Some clarification on why coming from the DRC to this specific refugee camp is a key risk factor for poorer outcomes would be helpful.

**Response**: This has been explained in the discussion paragraph that is highlighted in yellow

17. "It is among the few that provide quantitative analysis that prove the association between food insecurity and mental health concerns among children in refugee setting using validated tools" - I would query how this has been 'proved' from a single study, I think this could be better phrased in the text to not suggest a definite finding/pattern.

**Response**: This has been addressed in the 1st paragraph on page 5 (in the measurement tools section).

18. The methods section is generally clear, but the choice of convenience sampling should be discussed more thoroughly, including its limitations for generalizability.

**Response**: This has been addressed on Page 9 in the strengths and limitations.

19. Please specify whether the MINI-KID tool was culturally adapted or validated in this refugee setting to strengthen credibility

- **Response**: The MINI-KID has been used in Sub-Saharan African populations (https://doi.org/10.1186/s12888-025-07637-y) and among children in other refugee settings like Syrian refugee camps; however, it has not undergone a formal cultural validation in the Mahama refugee camp context. Prior to data collection, the tool was reviewed with local mental health professionals and refugee community workers to confirm appropriateness and comprehension of key terms.

20. The calculation of sample size is well described, but consider simplifying the explanation to improve readability for non-statistical readers

**Response**: This has been addressed in the sample size section on page 4

21. The Results section is comprehensive, but the large tables could be simplified or moved to supplementary materials for better flow.

**Response:** All the three (3) tables have been transferred to the “supplementary information” document but their legends are in the main manuscript as requested

22. The Discussion effectively relates findings to existing literature, but please reduce redundancy by avoiding repeating prevalence data multiple times

**Response:** This has been addressed

23. Consider expanding on the potential mechanisms linking food insecurity and depression in this setting to strengthen interpretation

**Response:** This has been addressed on page 11

24. The Conclusion is concise but could be more balanced by tempering strong policy recommendations with acknowledgment of the study’s design limitations.

**Response**: This has been addressed on page 12 in the conclusion section

25. Please check consistency in reporting prevalence (e.g., 69.4% vs 69.6% food insecurity) to avoid small discrepancies.

**Response**: Thanks for the key observation. This has been addressed

26. References are not cited in alphabetical order. let's address these

**Response:** I used the numbered format that’s why they aren’t alphabetically ordered
